# Interdisciplinary Case Study: How Mathematicians and Biologists Found Order in Cellular Noise

**DOI:** 10.1016/j.isci.2018.10.002

**Published:** 2018-10-06

**Authors:** Christopher Rackauckas, Thomas Schilling, Qing Nie

## Main Text

**Figure:**
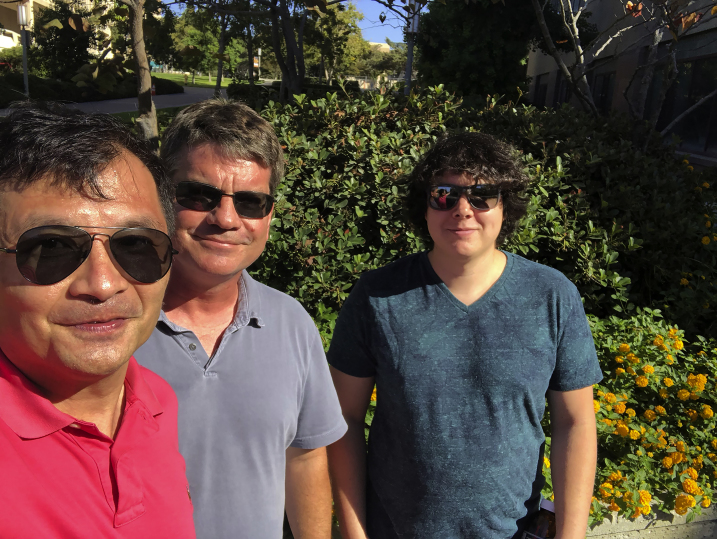
Nie, Schilling and Rackauckas outside of the labs.

During development, cells take cues from their immediate environment to decide their fate, but it is not always easy to “hear” the relevant information among all the genetic and molecular activities that are taking place. Cells will quiet this noise so that they can make accurate calls on how to behave. Using the zebrafish hindbrain as a testing ground, three University of California, Irvine (UCI), scientists—applied mathematician Christopher Rackauckas (now at MIT), developmental biologist Thomas Schilling, and mathematical biologist Qing Nie—identified a strategy called intermediate states that cells use to control noise. Their paper is one of the first examples of how a specific cellular protein can tone noise down to levels necessary for developmental activities.

This is the backstory for “Mean-Independent Noise Control of Cell Fates via Intermediate States,” published on April 10 in *iScience* (https://www.cell.com/iscience/fulltext/S2589-0042(18)30034-8#%20).

## Proximity

### The Paper Originated with a PhD Rotation

Rackauckas joined the Mathematical, Computational, and Systems Biology Gateway program at UCI, which is an interdisciplinary starter program that has first year PhDs do laboratory rotations in both a “dry” (mathematical or computational) and a “wet”(biological) laboratory. Rackauckas started to work on the zebrafish hindbrain system with a mathematical laboratory rotation focused on stochastic simulation with Qing Nie, and then rotated with Schilling's laboratory to clone crabp2b and learn/help with florescence lifetime imaging microscopy.

These two groups were collaborating on a project establishing the existence of noise control in the zebrafish hindbrain, but whereas the resulting publication could show noise control both in experiments and simulations, how such a property could arise was not understood. Rackauckas came up with an explanation and kept the project going in this explanatory direction.

## Language

### The Team Communicated Results by Treating Mathematical Statements like Biological Experiments

**Figure:**
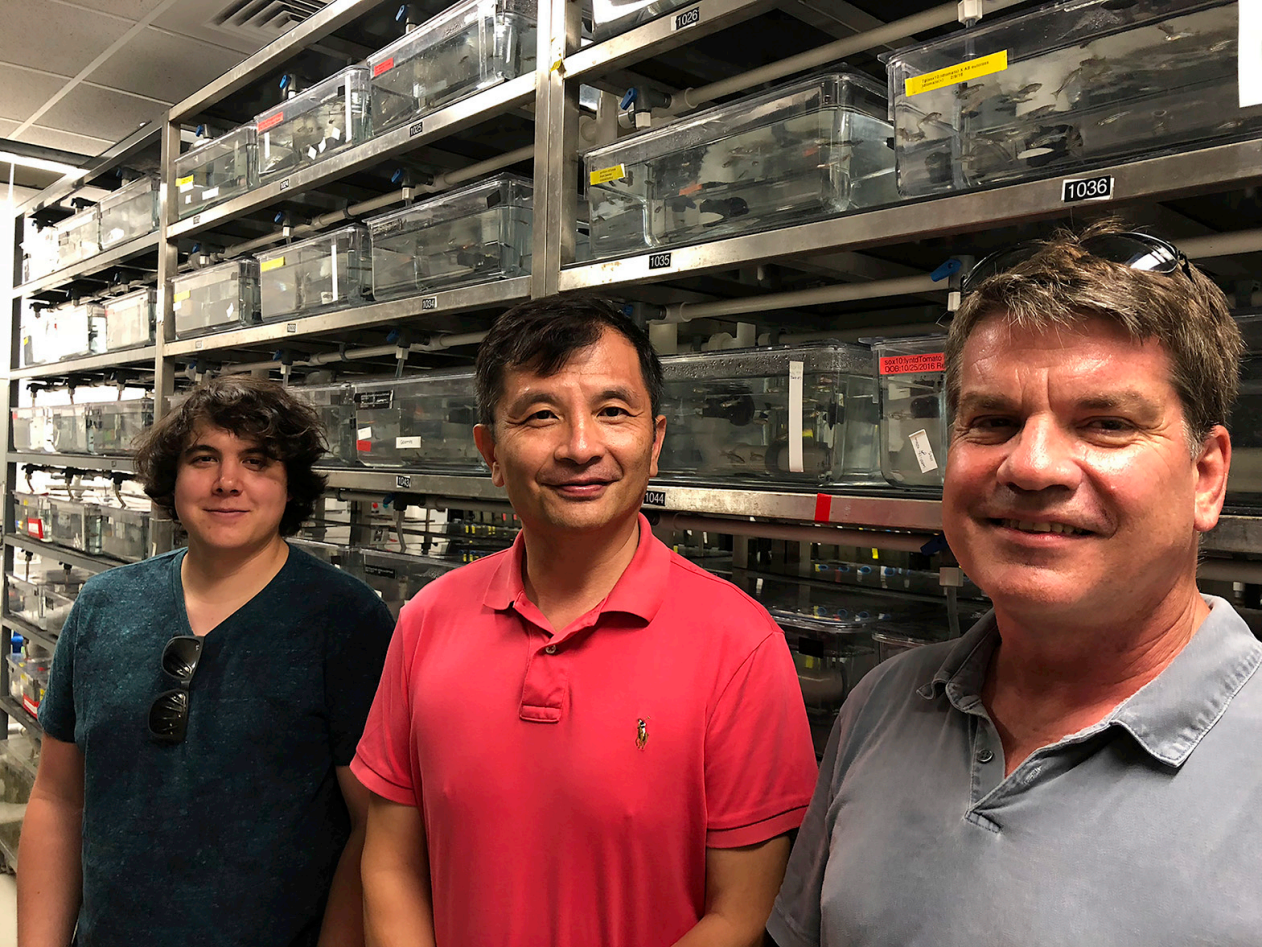
The researchers in the lab.

The central result of this work was the proof of (parameter-independent) mean-independent noise control on the various signaling network models. The issue was that said proof was baked in the language of stochastic differential equations, which is a very specialized branch of mathematics, but those results needed to be translated into usable heuristics to be applicable as biological knowledge.

After a few laboratory meetings and many discussions between the two sides, a methodology came along to treat the mathematical statement like a biological experiment. Instead of stating and proving a single fact in the mathematical style, the exposition that started to work followed a question-and-answer discovery. When Rackauckas would talk with Schilling, the discussion would always flow in the direction of questions like “crabp2b can upregulate its own production, what happens when you add feedback”? Eventually, the flow that was settled on for publication mirrored this discussion structure by presenting the simplified model and “testing” what happens as details are added, much like controlled biological experiments. We found that this allows one to get a feel for the robustness of the result without requiring someone to dig into the supplemental information.

## Methods

### Crossing Disciplines Meant Building New Programming Language

Interdisciplinary biological modeling seems to differ from “pure” mathematical biological modeling since having an expert in the system there at each discussion drives you toward a form of realism that pushes mathematical and computational methods to their limits. One of the difficulties of this project was demonstrating the spatial effects since the original MATLAB code and their Euler-Maruyama discretizations were not scaling to the stiff stochastic partial differential equations with downstream Hox/Krox effects with the associated feedbacks. Instead of simplifying the model, it instead became a simultaneous project to build new simulator codes capable of such scale. The resulting DifferentialEquations.jl ecosystem in the Julia programming language and publications in the journal *Adaptive Methods for Stochastic Differential Equations* were driven by the need to solve the model without extra simplifications.

Having an expert in the biological system in the room at each discussion drove the project towards a form of realism that pushed mathematical and computational methods to their limits.

## Governance

### Joint NIH Grants Helped Fund the Project

The laboratories of Nie and Schilling are linked by joint NIH grants on mathematical models and explanations of zebrafish hindbrain and craniofacial development. This has had a profound effect on the “Nie Lab” since, although it is centered in a traditional mathematics department, it is not structured like the traditional adviser/advisee pairing of the discipline and functions more like a scientific laboratory. This interdisciplinary work has had a profound effect on Rackauckas's career trajectory since the development of DifferentialEquations.jl, with its focus on scalable stochastic modeling, has gained wide adoption both in biological modeling and in other fields.

## Publication

### The Challenge of Reaching Biologists (and Mathematicians) with Stochastic Differential Equations

**Figure:**
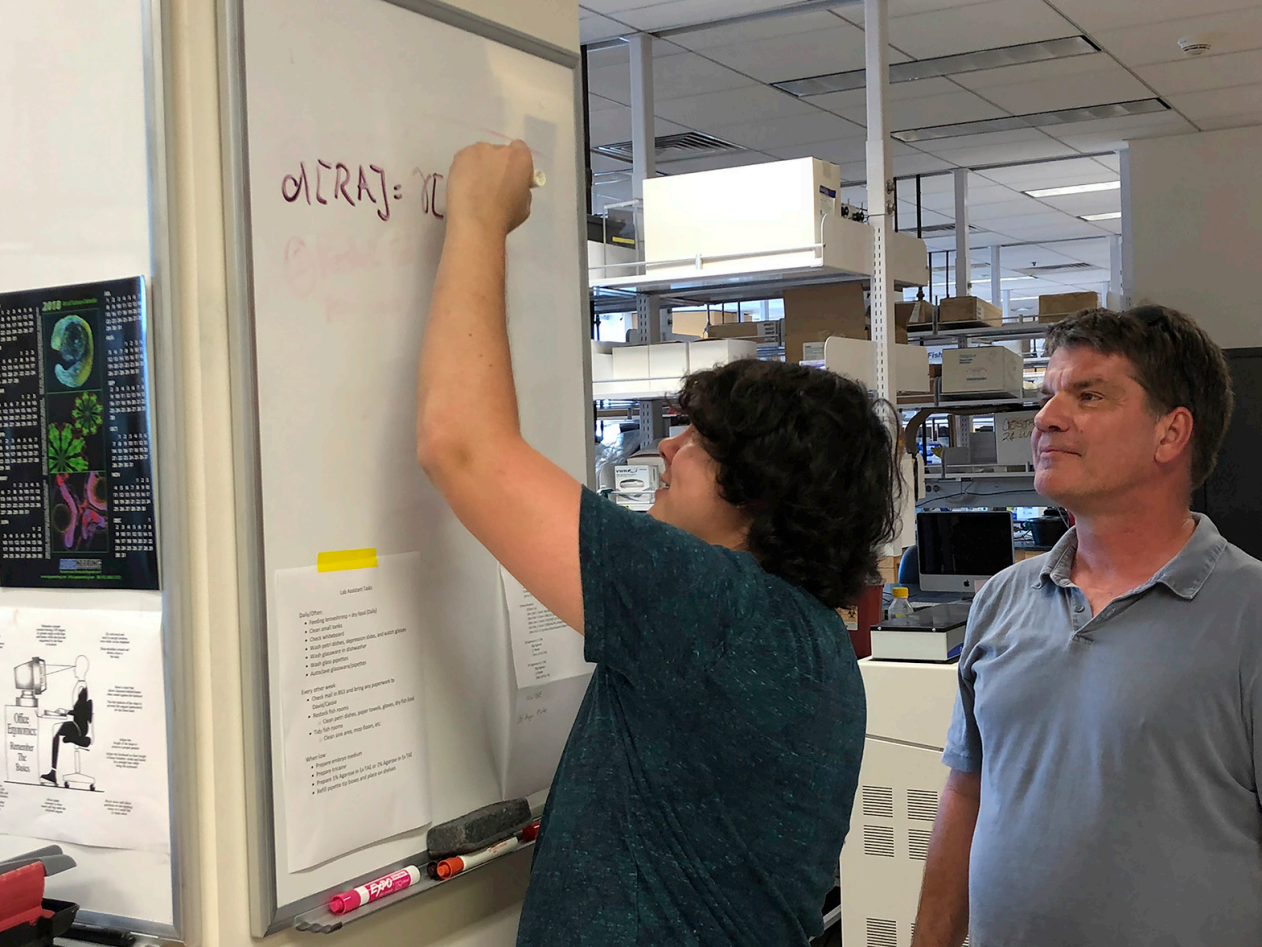
The language of differential equations.

We felt that the result is a useful heuristic for biologists to reason about noise control and help give an explanation as to why certain network structures exist. The issue is that, although the result is of biological importance, the language of stochastic differential equations is not standard for biologists (or mathematicians). The discussion of “randomness” needed the mathematics to be well grounded, but we wanted the end result to be more toward building heuristic knowledge of noise in interactions so that a biologist could make statements like “I would expect Protein A to increase the randomness in the concentration of Protein B.”

The end result needed to build heuristic knowledge of noise in interactions, so that a biologist could make statements like ‘I would expect Protein A to increase the randomness in the concentration of Protein B.

Thus the publication was developed in a way that has the mathematical details as supplemental to the numerical experiments, which show the resilience of the effect to keep the focus on the biological outcomes and not the method used for the explanation. This put many constraints on the publication venue since mathematical biology journals like to see the mathematical results as front and center, whereas the mode of explanation being entirely math was not fit for standard biological journals or even many systems biology journals.

### Final Thoughts

Interdisciplinary research is difficult because nobody understands the full story, that is why you are collaborating! The issue that then arises during the publication process is that no reviewer will be an expert in both the methods and application you are looking at. We hope that channels that specifically publish interdisciplinary research can be better suited for this kind of work.

Interdisciplinary research is difficult because nobody understands the full story, that is why you are collaborating!

The interdisciplinary case study series highlights how research teams have overcome the barriers related to proximity, language, methods, governance, publication, and funding that arise when bringing people together from two or more unrelated fields. Read more backstories and get inspired at *iScience*.

